# 0531. Cumulative effects of negative energy balance on myocardial deformity and diastolic function during the first week of ICU: a pilot study

**DOI:** 10.1186/2197-425X-2-S1-P31

**Published:** 2014-09-26

**Authors:** A Gómez Blizniak, DF Matallana Zapata, M Ruiz Bailén, A Morante, E Castillo-Lorente, MD Pola de Gallego, F Ruiz Ferrón, J La Rosa, AM Castillo, L Rucabado Aguilar

**Affiliations:** Complejo Hospitalario de Jaén, Critical Care Unit, Jaén, Spain

## Objectives

- To evaluate whether a greater negative energy balance (NEB) accumulated during the first week of ICU correlates with worsening in longitudinal Strain (LS) and diastolic function (DF).
- To evaluate whether improvement in the nutritional status (NS) correlates with improvement in LS and DF.

## Methods

We made an observational, analytical, prospective, longitudinal pilot study.

***Dependents variables:***

***LS***, echocardiographic parameter used to assess myocardial deformity (contraction). We considered as an improvement an increase ≥ 10%.

***E/é ratio***, parameter used to assess DF. A reduction of E/é ratio ≥ 10 % was considered DF improvement.

***I. V,:***

***NEB during the first week of admision***.

***Improvement in the NS***: assessed by an increase in at least one level of prealbumin nutritional scale (PNS) after 10 days of receiving 100 % of estimated energy (EE) requirements (H. Benedict).

(PNS: Normal>18 md/dl, mild undernutrition: 17.9-15 , moderate: 14,9-10 severe < 10).

Convenience nonprobability sample.

**S. analysis:** The results were expressed as means with their ST deviations, %. Linear regression (LR) and Fisher test (FT) were used to analyze possible statistics associations, expressed with their CI and p values.

**TTE were performed to** patients admitted from July to October, 2013, in the first 24 h of admision, at 7th and 10th days of receiving enteral and/or parenteral nutrition with 100 % of EE. Acoustic catches are done in HQ digital format, f.r.> 100 Hz, for further analysis “of line" of LS. (Blind analysis).

**Exclusion crit.**: nephrotic syndrome, cirrhosis, chronic renal and HF.

PCR, MV (PEEP), PVC were recorded.

## Results

10 patients, 60 % male, mean age: 54 (27-75). 30 % normal NS, 30 % mild, 10% moderate and 30 % severe undernutrition. 40 % traumatic and 30 % spontaneous ICH , 10% thoracic trauma , 10% cardiac arrest and 10 % septic shock . 70 % required MV, 20 % norepinephrine.

KS test: p = 0.595. We observe a tendency to an inverse relationship (**p = 0.375**, r = - 0.315, N = 10) between NEB and LS but not s. significant. 40% of those who had improvement in at least 1 level of the PNS showed a 10% increase in LV LS at 10 days receiving 100% EE (FT: **p = 0.714, OR: 0.667 , 95% CI : 0.025 to 18.059**).

As in the Hammer et al study [1], in which acute progressive caloric restriction in young healthy men correlated with impaired DF, we observed a direct relationship (r = 0.462 , p = 0.434 , N = 5) between NEB and E/é, but not s. significant. The 50% who had an improvement in NS showed a 10% reduction in E /é (FT: **p = 1.00, OR 1.00 , 95% CI : 0.03 to 29**).

**Figure 1:**
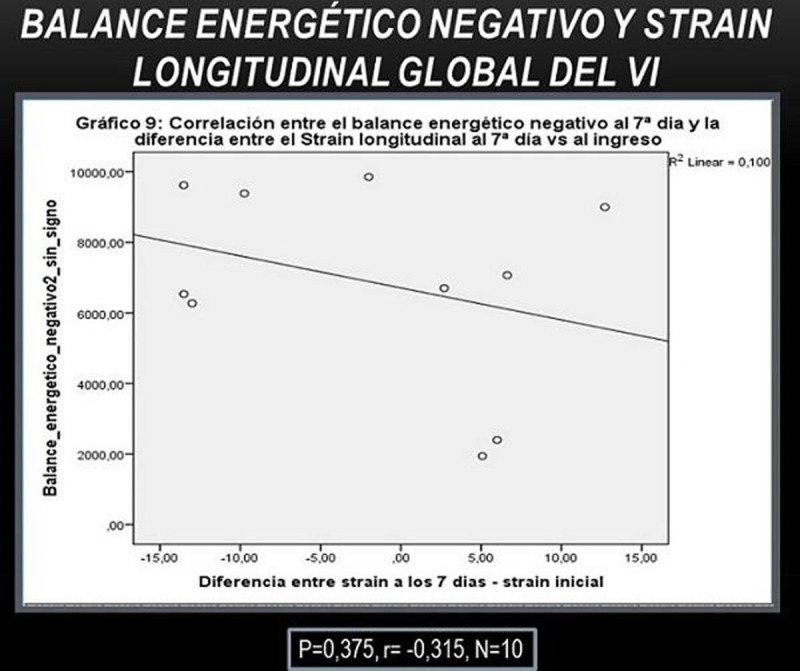
Longitudinal strain AND BEN

**Figure 2:**
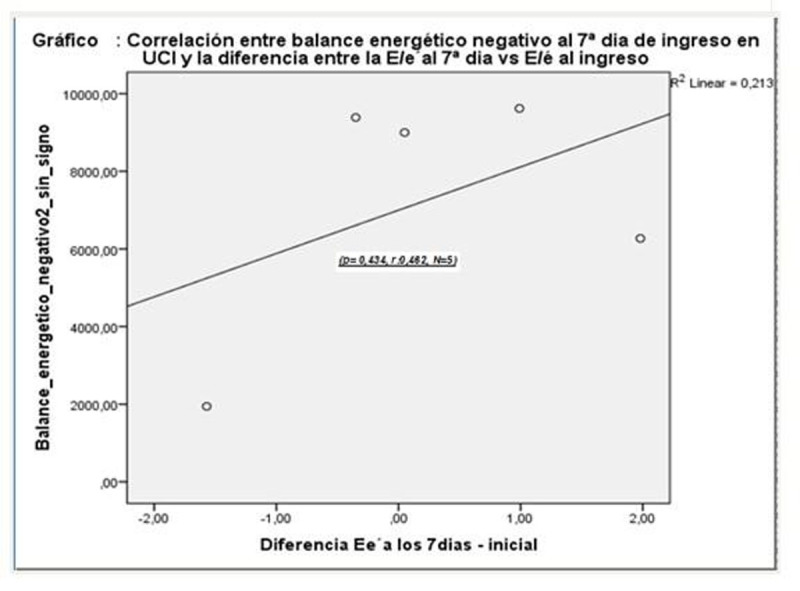
BEN AND DF

**Figure 3:**
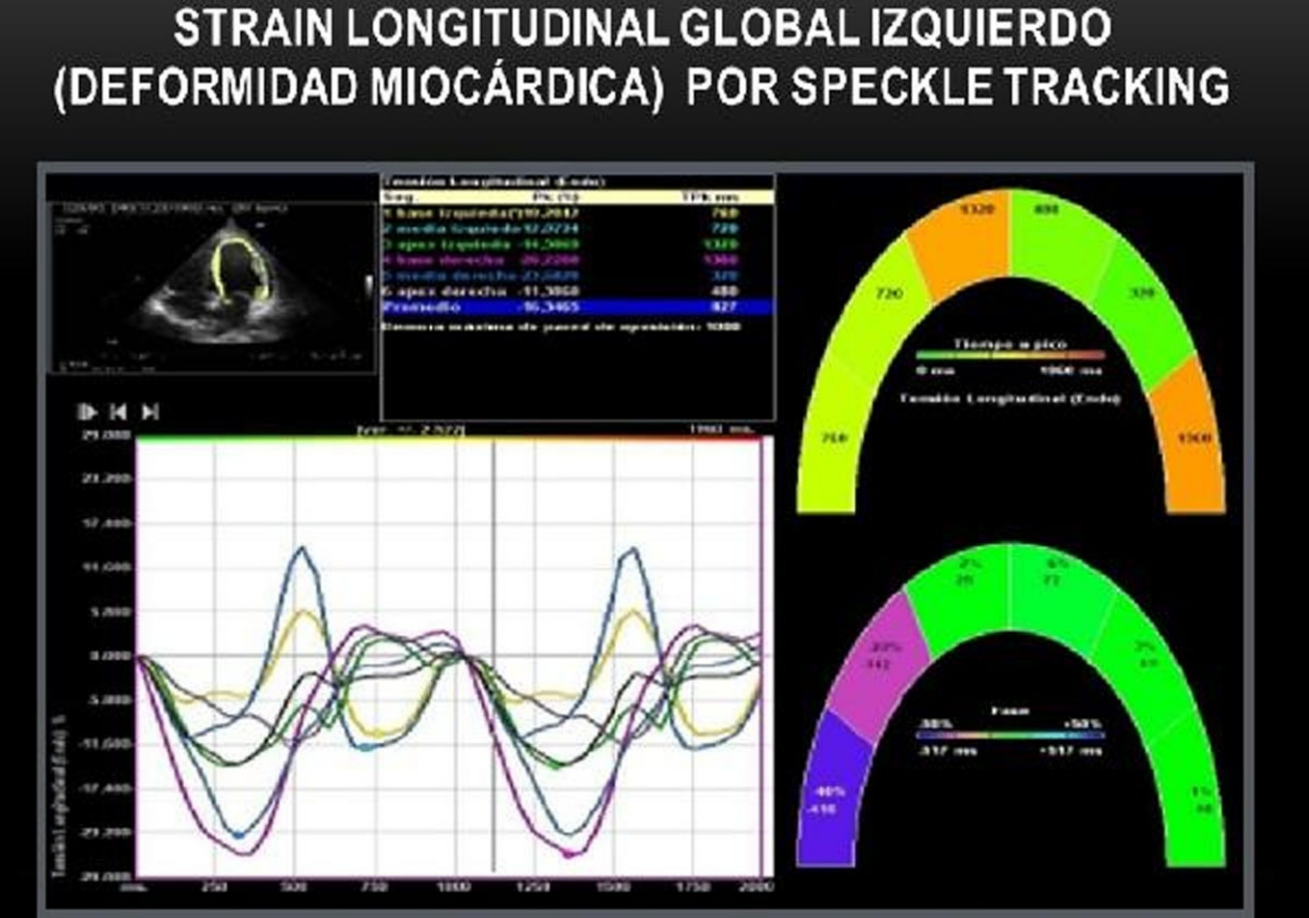
Global LS

## Conclusions

Patients with higher cumulative NEB during the first week of ICU had a decrease in LS and an increase in E/é but not s. significant. Given the limitations of this research (being a pilot study of a topic not addressed in ICU with few patients) should be carried further study with sufficient power to test this hypothesis.
